# Genetic Landscape of a Pleural Mesothelioma in a Child Affected by NF2-Related Schwannomatosis

**DOI:** 10.3390/ijms26146848

**Published:** 2025-07-16

**Authors:** Marzia Ognibene, Gianluca Piccolo, Marco Crocco, Marco Di Duca, Antonio Verrico, Marta Molteni, Ferruccio Romano, Valeria Capra, Andrea Rossi, Federico Zara, Patrizia De Marco, Claudia Milanaccio

**Affiliations:** 1 U.O.C. Genetica Medica, IRCCS Istituto Giannina Gaslini, 16147 Genova, Italy; marcodiduca@gaslini.org (M.D.D.); federicozara@gaslini.org (F.Z.); patriziademarco@gaslini.org (P.D.M.); 2 U.O.S.D. Neuro Oncologia, IRCCS Istituto Giannina Gaslini, 16147 Genova, Italy; gianlucapiccolo@gaslini.org (G.P.); antonioverrico@gaslini.org (A.V.); martamolteni@gaslini.org (M.M.); claudiamilanaccio@gaslini.org (C.M.); 3 Dipartimento di Neuroscienze, Riabilitazione, Oftalmologia, Genetica e Scienze Materno-Infantili, Università Degli Studi di Genova, 16132 Genova, Italy; 4 U.O.C. Gastroenterologia Pediatrica, IRCCS Istituto Giannina Gaslini, 16147 Genova, Italy; marcocrocco@gaslini.org; 5 U.O.C. Genetica Clinica, IRCCS Istituto Giannina Gaslini, 16147 Genova, Italy; ferruccioromano@gaslini.org (F.R.); valeriacapra@gaslini.org (V.C.); 6 U.O.C. Neuroradiologia, IRCCS Istituto Giannina Gaslini, 16147 Genova, Italy; andrearossi@gaslini.org

**Keywords:** NF2-related schwannomatosis, neurofibromatosis type 2, pediatric pleural mesothelioma, chromosomal aberrations, cancer-related genes

## Abstract

We report the first case of pleural mesothelioma (PM) occurring in a child affected by NF2-related schwannomatosis (NF2-SWN) and without any history of environmental exposure to asbestos. Mesothelioma is a rare secondary tumor in brain cancer patients and the association with NF2-SWN has been described only in a few anecdotal cases and never in the pediatric field. NF2-SWN is an autosomal dominant disease caused by inactivating germline mutations of the *NF2* tumor suppressor gene, one of the most common mutations associated with human primary mesothelioma too. By MLPA assay, array-CGH analysis, and NGS on blood and tumor DNA, we determined the mutation profile of this rare *NF2*-driven PM and we identified several atypical chromosomal aberrations in tumor cells, suggesting a different genomic signature between pediatric and adult mesothelioma.

## 1. Introduction

Mesothelioma emerges principally from the mesothelial cells belonging to the serosal membrane that covers the pleural, peritoneal, and pericardial cavities and can be divided into benign and malignant types [1]. Pleural mesothelioma (PM) is an aggressive rare disease with an incidence in Italy of about 2.46 per 100,000 inhabitants, and it is the most common form of mesothelioma, representing about 80% of cases of this tumor. Depending on the histology, from better to worse prognosis, it is classified as epithelioid, biphasic, or sarcomatoid. Pleural mesothelioma is mainly linked to industrial pollutants and mineral fiber exposure, with approximately 85% of cases linked to asbestos. Though asbestos is certainly the largest and most well-known cause of mesothelioma, roughly 20% of patients do not have any known exposure [2]. Genetic analysis and other studies have led to the suspicion that genetic predisposition and radiation therapy are other possible culprits [3,4].

Less than 1% of patients affected by PM without a known exposure carry a specific inherited genetic mutation. These inherited mesotheliomas appear at a younger age and demonstrate no sex or specific anatomic site predilection. Additionally, the possible presence of other concurrent cancers, in particular melanomas and renal cell carcinomas, suggests a broader cancer predisposition syndrome [5]. For the successful development of diagnostic, prognostic, and personalized therapies, it is crucial to understand the genetic alterations that drive PM. Since PM is a rare disease, genomic studies are limited and involve only a small number of cases. Loss-of-function mutations in *BAP1*, *NF2*, and *CDKN2A* have been previously described in PM [6,7], and other studies have reported copy gains and copy losses involving many different regions of the genome [8]. Recent whole-genome sequencing analysis identified new significantly mutated genes in PM, including *RDX*, *PIK3C2B*, *TAOK1*, *TP53*, *DDX3X*, *SETD2*, *SF3B1*, and *TRAF7* [6,9].

Mesothelioma is extremely rare in children, usually not related to asbestos exposure, and with some clinical differences with the adult forms. Furthermore, the prognosis of pediatric mesothelioma appears to be more favorable than its adult analog. The median age at onset is 13.4 years [1].

NF2 (Neurofibromatosis type 2)-related schwannomatosis (NF2-SWN) is an autosomal dominant disorder whose characteristic features include multiple nervous system tumors (vestibular schwannomas, intracranial meningiomas, spinal ependymomas, and peripheral nerve tumors), ocular abnormalities, and skin lesions [10]. Tumors associated with NF2-SWN are caused by inactivating mutations or loss of both alleles (constitutional and somatic mutations) of the *NF2* tumor suppressor gene. *NF2* expresses the Merlin protein, an ERM (ezrin–radixin–moesin)-like molecule that interacts with cell surface proteins involved in cytoskeletal dynamics and in the regulation of ion transport [11]. *NF2* mutations are also detectable in sporadic schwannomas, in meningiomas, and, peculiarly, in sporadic malignant mesotheliomas, indicating that the *NF2* gene may be a critical growth regulator for all these cell types. Even if mesothelioma is not a distinctive feature of NF2-SWN, a high prevalence of mesothelioma in NF2-SWN could nevertheless escape detection because both diseases are rare [12].

It has been suggested that NF2-SWN patients could be at increased risk of developing mesothelioma, but, actually, patients with inherited NF2-SWN rarely develop mesothelioma as a second cancer and the few reported cases all happened in adult age [13,14,15].

We report here the first known case of a pediatric patient diagnosed with syndromic NF2-SWN at the age of one year, who twelve years later developed a PM. We identified a germline *NF2* loss-of-function mutation and, in PM, a second hit, the loss of the *NF2* locus on chromosome 22, resulting in *NF2* bi-allelic inactivation, together with many other numerical chromosomal aberrations, some of which are unusual for a PM genetic profile.

## 2. Results

### 2.1. Patient Information

**Figure 1:**
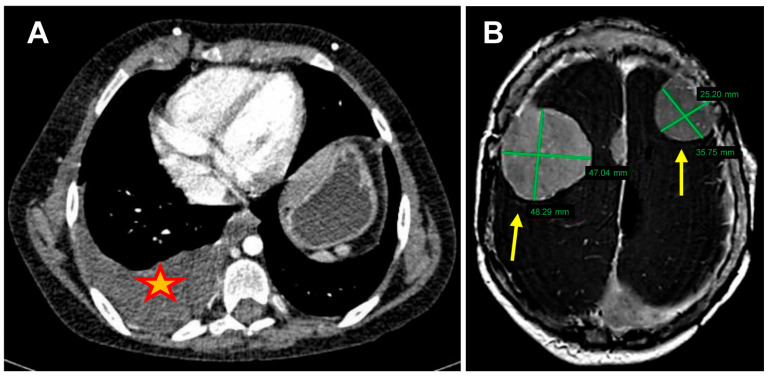
(**A**) Computed Tomography scan of abdomen (transverse plane), showing pleural thickening and effusion (yellow star); (**B**) brain MRI (T1 post-contrast, axial), showing rapidly increasing bilateral recurrent meningiomas (yellow arrows).

A one-year-old boy was diagnosed with severe NF2-related schwannomatosis and treated with several neurosurgical exereses (cerebral and spinal meningiomas and vestibular schwannomas) and with multiple chemotherapy lines (hydroxycarbamide, bevacizumab, and sirolimus), the latter all proving ineffective. After twelve years, he developed pleural effusion and weight loss. The CT scan showed the thickening of the right pleura and multiple peritoneal nodules (Figure 1A). The cytological examination on thoracentesis diagnosed an epithelioid PM that followed a mild course, giving an overall survival of about one year; then the patient died because of rapidly increasing meningiomas related to NF2-SWN (Figure 1B). Importantly, there was no tumor family history and no environmental exposure to asbestos contamination.

### 2.2. Molecular Diagnosis of NF2-Related Schwannomatosis

**Figure 2:**
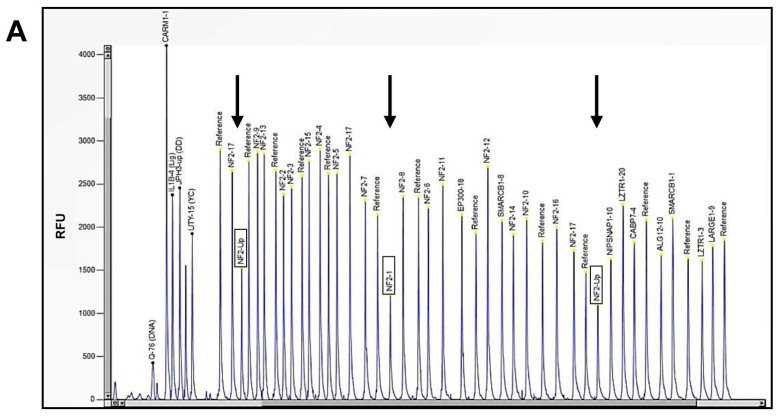

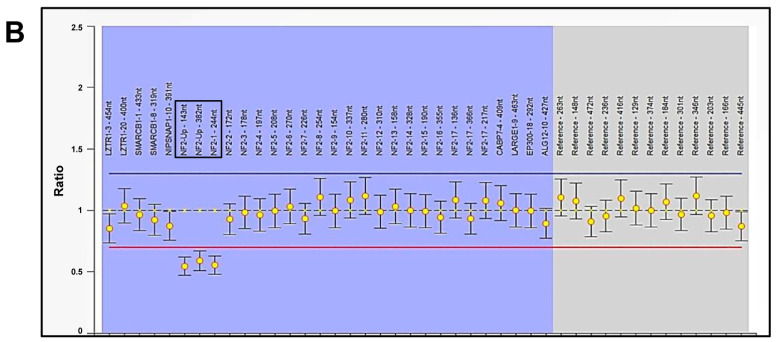
MLPA analysis of patient’s genomic DNA: (**A**) the capillary electrophoresis pattern identified lower peaks for the 5’UTR and exon 1 of *NF2* (indicated by black arrows) (RFU: relative fluorescence units); (**B**) ratio chart of the MLPA results, with the blue line indicating the threshold to identify duplication of genetic material and the red line identifying the deletion. *NF2* 5’UTR and exon 1 heterozygous deletion were visible under the red line.

No pathogenic single-nucleotide variants (SNVs) and no small insertions/deletions were identified through NGS of three NF2-SWN-related genes (*NF2*, *LZTR1*, and *SMARCB1*) in the genomic patient’s DNA. Nevertheless, by MLPA assay (Multiplex Ligation-dependent Probe Amplification), we discovered a heterozygous deletion of the *NF2* gene (NM_000268.3) encompassing the 5’UTR (414 bps before the start codon) and exon 1 (Figure 2A,B), thus confirming the clinical diagnosis.

### 2.3. Genomic Profile Analysis of Mesothelioma Cells by Array-CGH

**Figure 3:**
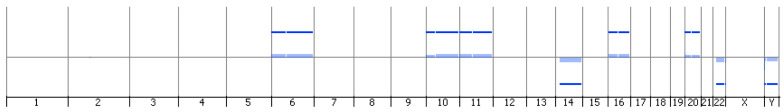
Genomic profile obtained by array-CGH on DNA extracted from mesothelioma biopsy of the patient (effusion cells), displaying duplication of chromosomes 6, 10, 11, 16, and 20 and deletion of chromosomes 14, 22, and Y.

Since PM is a tumor frequently characterized by typical chromosomal abnormalities [16], the DNA from the patient’s PM effusion cells was analyzed by a-CGH. We found a gain of whole chromosomes 6, 10, 11, 16, and 20 and loss of whole chromosomes 14, 22, and Y (Figure 3).

### 2.4. Genomic Profiling of Mesothelioma Cells by NGS Analysis

To assess the complete mutational profile of the tumor, we performed NGS on DNA extracted from mesothelioma effusion cells using a commercial NGS panel covering 501 cancer-associated genes. The analysis did not find any pathogenic SNVs on cancer-related genes, but it did find many copy number variations (CNVs) that substantially confirmed the a-CGH results (Table 1) and the somatic loss of the *NF2* locus. The *NF2* second hit confirmed the role of this tumor suppressor gene as a driver for PM. The analysis also identified co-occurring mutations commonly present in tumors, which may activate complementary oncogenic pathways, such as *PARP2*, *FANCM*, *RAD51B*, *MLH3*, *DICER1*, and *XRCC3* on chromosome 14q and *MAPK1*, *SMARCB1*, *CHEK2*, *NF2*, and *EP300* on chromosome 22q, whose function, apart from *NF2*, is, so far, not for all clearly associable with PM.

## 3. Discussion

Both NF2-SWN and PM are related to *NF2* dysfunction; consequently, it may be plausible that NF2 patients are at increased risk of PM. However, to date, very few cases describing patients diagnosed with both NF2 and malignant mesothelioma have been reported by the literature [13,14,15], and they were all adults.

*NF2* mutations are linked to peritoneal and pericardial mesothelioma too. *NF2* mutations were identified in 21–35% of cases of peritoneal mesothelioma [17]. Primary pericardial mesotheliomas are extremely rare, accounting for <1% of all mesotheliomas, and they share similar genomic aberrations with peritoneal mesotheliomas, being driven by several genes including *NF2* [18].

We describe here the first case of a pediatric patient affected by NF2-SWN, who twelve years later, at the age of 13, developed a PM. Furthermore, he was never subjected to asbestos contamination and he had no tumor family history. We identified a pathogenic heterozygous deletion of *NF2* in blood DNA and the concomitant loss of the *NF2* locus in the tumor through the loss of the whole chromosome 22, demonstrating that PM was driven by the bi-allelic loss of function of *NF2*. In adults, *NF2* inactivation in mesothelioma cells is considered a late event that may result in an aggressive phenotype [19]. Here we propose that in pediatric mesothelioma *NF2* inactivation could instead represent an early driver event.

The NGS analysis of tumor DNA showed the presence of several CNVs, substantially recalling the same numerical chromosomal aberrations identified by a-CGH. Apart from the loss of chromosomes 14 and 22, the other found chromosomal aberrations are not typical of mesothelioma [8,16,20]. In particular, mesothelioma is mainly characterized by the loss of the p or q arm of chromosomes rather than gain [17], while our patient had only entire affected chromosomes and much more gained than lost ones. It is conceivable that the loss of *NF2* together with the deletion of other tumor suppressor genes or with the duplication of oncogenes contained in the chromosomes, respectively, found lost or gained in this patient, and, to date, not associated with mesothelioma, may have acted collectively in our patient.

In particular, NGS analysis highlighted ten cancer-related genes (*PARP2*, *FANCM*, *RAD51B*, *MLH3*, *DICER1*, *XRCC3*, *MAPK1*, *SMARCB1*, *CHEK2*, and *EP300*), besides *NF2*, whose loss is still not clearly proven to be related to PM. While the correlation of PM with MAPK pathways genes such as *MAPK1* or with *EP300* is quite well described [20,21], for *CHEK2*, *FANCM*, *XRCC3*, and *SMARCB1* some suggestion has been proposed [21,22,23,24]. Surely interesting and not yet reported as associated with PM are *RAD51B* and *MLH3*, both essential for DNA mismatch repair [25,26].

Several studies have investigated genes and genetic mechanisms associated with PM, employing various kind of technologies such as Sanger sequencing, genome-wide association (GWAS), and whole-genome sequencing. In adult PM, the oncogenic pathways of mTOR, Hippo (*NF2*), and p53 were mainly associated with the tumor. Alterations in *BAP1*, *CDKN2A*, *CDKN2B*, *NF2*, *MTAP*, *TP53*, and *SETD2* occurred with an incidence of at least 10% [17]. Recurring chromosomal changes included partial deletions of 1p and 3p and monosomy of 14, 18, 19, and 22. Pediatric PM is an extremely rare disease, with very few cases published in the literature. It appears to be a different entity than its adult counterpart, since it is never associated with asbestos or radiation exposure [27]. Recently, pediatric malignant peritoneal mesothelioma cases have been genetically studied, with alterations observed in *ALK*, *EWSR1*, *FUS1*, *YY1*, or in *AURKA*, *AURKC*, *HLA-1B*, *ZNF-217*, *OR5F1*, and *MEN1* genes, but not in *BAP1* [28,29].

While most PMs arise from exposure to environmental carcinogenic factors with a long latency period of about 30–60 years, a small percentage of cases occur spontaneously from somatic or germline variants. Adult PM related to inherited germline variants of *BAP1* occurs at a younger age, 55 versus 72 years [30]. As for *NF2*, although many NF2 patients reach the age of morbidity for mesothelioma onset, the development of mesothelioma has not specifically been reported, suggesting that *NF2* germline mutation is unlikely to be a predisposing condition for familial PM development like *BAP1*.

All the cancer genes here identified or even others not yet described could consequently represent possible genetic risk factors for juvenile PM occurrence in the absence of environmental contamination. Future additional investigations and functional studies are needed to understand which genes could be preferentially linked with the malignant evolution of pediatric pleural mesothelioma.

## 4. Materials and Methods

### 4.1. Sample Collection

Genomic DNA from peripheral blood lymphocytes and PM DNA from pleural effusion cells of the patient were extracted using the QIAsymphony DNA kit (Qiagen, Hilden, Germany) and the NucleoSpin Tissue kit (Macherey-Nagel, Düren, Germany), respectively, according to the manufacturer’s instructions. Written informed consent was obtained from the patient’s parents in accordance with the Declaration of Helsinki.

### 4.2. Targeted Next-Generation Sequencing (NGS) of NF2-SWN-Related Genes

Genomic DNA was analyzed for the sequencing of three NF2-SWN-related genes. Given the clinical overlap between Neurofibromatosis type 2 and schwannomatosis, we developed an NGS customized panel using the Ion AmpliSeq™ Designer v6.13 algorithm provided by Thermo Fisher Scientific (Carlsbad, CA, USA) in order to target the entire coding sequence (CDS) and 10 bases of the adjacent intronic regions of NF2 (NM_000268.3), LZTR1 (NM_006767), and SMARCB1 (NM_003073.3). The primers pools were composed of 117 amplicons showing the following features: 100% total coverage, 0% missed regions, and amplicon range of 125–275 bps. Libraries were prepared starting from 10 ng of genomic DNA using the AmpliSeq Library Kit 2.0 (Thermo Fisher Scientific), according to the manufacturer’s instructions. The final concentration of the library was evaluated with a Qubit^®^ 2.0 Fluorometer using the Agilent High Sensitivity DNA Kit (Thermo Fisher Scientific). Template preparation and chip loading were performed on the Ion Chef System (Thermo Fisher Scientific). The sequencing was performed on Gene Studio S5 (Thermo Fisher Scientific) using a 510 Ion Chip. Base calling was generated by Torrent Suite 3.0 software (Thermo Fisher Scientific), using tmap-f3 on the Ion Torrent server for further analysis. Bam files were analyzed by Ion Reporter Software v.5.16 (https://ionreporter.thermofisher.com/ir/, accessed on 10 March 2025).

### 4.3. Multiplex Ligation-Dependent Probe Amplification Analysis (MLPA)

Genomic DNA was analyzed with the SALSA MLPA probemix P044-B3 NF2 (MRC-Holland, Amsterdam, The Netherlands) to identify single- and multi-exon deletions/duplications inside the *NF2* gene. Results obtained by ABI Prism 3130 Genetic Analyzer (ThermoFisher Scientific) were analyzed with Coffalyser Net software, version v.240129.1959 (MRC Holland). The mean cut-off for the normalized peak height ratio of the patient to the control sample was less than 0.7 in case of exon deletions and more than 1.30 in case of exon duplications.

### 4.4. Genomic Profile Analysis by Array-Comparative Genomic Hybridization (a-CGH)

DNA from PM effusion cells was tested by high-resolution oligonucleotide a-CGH using the 4 × 180 K Kit (Agilent Technologies, Santa Clara, CA, USA) with a mean resolution of 25 kb. The images produced by each hybridization were processed using Agilent Feature Extraction 10.5 software, and the obtained data were analyzed using Genomic Workbench 7.0.40 software (Agilent), as previously described [31].

### 4.5. Targeted NGS of Cancer-Related Genes

Comprehensive molecular profiling of tumor DNA was performed using Oncomine Comprehensive Assay Plus (OCA Plus, Thermo Fisher Scientific), which covers 501 cancer-associated genes. The panel detects telomeric allelic imbalance (TAI), large-scale state transitions (LST), microsatellite instability (MSI), tumor mutational burden (TMB), and HRD (Homologous Repair Deficiency) all in one workflow. In general, multiplex PCR amplification was conducted using a nucleic acid concentration of approximately 20 ng as input. Manually prepared libraries were loaded according to the manufacturer’s instructions and quantified using the Ion Library TaqMan Quantitation kit (Thermo Fisher Scientific). All libraries were adjusted to 50 pM before template preparation and 550™ chip loading using the Ion Chef™ System according to the manufacturer’s instructions. Sequencing was performed using the Ion GeneStudio™ S5 Prime Sequencer (Thermo Fisher Scientific). Libraries were loaded on 550^TM^ Chips (Thermo Fisher Scientific) using the Ion Chef™ liquid handler in order to obtain a median coverage >1000× and to detect variants with a minor allelic frequency (MAF) of 1%.

### 4.6. Bioinformatics Analysis

BAM files were analyzed using Ion Reporter™ Software version 5.20 (Thermo Fisher Scientific) with the Oncomine Comprehensive Plus-w3.1-DNA and Fusions-Single Sample workflow. Variants were ultimately classified using the tier-based system defined by a joint consensus recommendation of the Association for Molecular Pathology, American Society of Clinical Oncology, and College of American Pathologists. Copy number estimates were made using a proprietary VCIB (Variability Control Informatics Baseline) algorithm. The VCIB informatics baseline was created using at least 48 diverse samples with at least 6 normal samples. The MAPD metric is a measure of read coverage noise detected across all amplicons in a panel. Higher MAPD typically translates to lower coverage uniformity. Lower coverage uniformity can result in missed or erroneous CNV calls. MAPD score is viewable in a downloadable VCF file or in the review of the analysis results of a single-sample extended analysis. To make a CNV call, the following criteria must be met: MAPD 2; *p*-value < 0.85.

## 5. Conclusions

In conclusion, we report here the genetic study of the first known pediatric case of a patient affected by both NF2-SWN and PM, suggesting the possible existence of a different genomic signature compared to the adult analog. Indeed, our patient did not display the genetic mutations classically associated with adult PM, apart from the loss of the *NF2* locus, causative of the severe schwannomatosis, and the loss of chromosomes 14 and 22. Further studies are therefore needed to determine which genes could be specifically related to pediatric PM in order to address targeted molecular-based therapeutic approaches.

## Figures and Tables

**Table 1:** Genes and chromosomes displaying CNVs in tumor DNA.

Locus *	Filter	Variant Class	Variant ID	Cytoband and Copy Number **
chr6:350767	GAIN	CNV	ARM 6p	6p25.3p12.1 (350,767–55,739,590) × 3
chr6:62442567	GAIN	CNV	ARM 6q	6q11.1q27 (62,442,567–170,627,672) × 3
chr10:323466	GAIN	CNV	ARM 10p	10p15.3p11.22 (323,466–32,856,813) × 3
chr10:43292608	GAIN	CNV	ARM 10q	10q11.21q26.2 (43,292,608–129,902,370) × 3
chr11:403974	GAIN	CNV	ARM 11p	11p15.5p11.12 (403,974–50,003,230) × 3
chr11:55032426	GAIN	CNV	ARM 11q	11q11q24.3 (55,032,426–130,776,619) × 3
chr14:20248397	LOSS	CNV	ARM 14q	14q11.2q32.33 (20,248,397–105,246,558) × 1
chr14:20811781	LOSS	CNV	*PARP2*	14q11.2 (20,811,781–20,825,977) × 0.942029
chr14:45605157	LOSS	CNV	*FANCM*	14q21.2 (45,605,157–45,669,234) × 1.05797
chr14:68290164	LOSS	CNV	*RAD51B*	14q24.1 (68,290,164–69,061,406) × 1
chr14:68290164	LOSS	LOH	*RAD51B*	14q24.1 (68,290,164–69,061,406) × 1
chr14:75483761	LOSS	CNV	*MLH3*	14q24.3 (75,483,761–75,516,400) × 0.971014
chr14:95556791	LOSS	CNV	*DICER1*	14q32.13 (95,556,791–95,599,859) × 1.07246
chr14:104165043	LOSS	CNV	*XRCC3*	14q32.33 (104,165,043–104,177,450) × 1.05797
chr16:336630	GAIN	CNV	ARM 16p	16p13.3p11.2 (336,630–31,092,113) × 3
chr16:47697595	GAIN	CNV	ARM 16q	16q12.1q24.3 (47,697,595–89,883,082) × 3
chr20:391069	GAIN	CNV	ARM 20p	20p13p11.21 (391,069–25,187,259) × 3
chr20:30954155	GAIN	CNV	ARM 20q	20q11.21q13.33 (30,954,155–62,595,178) × 3
chr22:22123473	LOSS	CNV	*MAPK1*	22q11.21 (22,123,473–22,162,093) × 1.07246
chr22:24129273	LOSS	CNV	*SMARCB1*	22q11.23 (24,129,273–24,176,467) × 0.913043
chr22:29083868	LOSS	CNV	*CHEK2*	22q12.1 (29,083,868–29,130,729) × 1
chr22:29083868	LOSS	LOH	*CHEK2*	22q12.1 (29,083,868–29,130,729) × 1
chr22:29999923	LOSS	CNV	*NF2*	22q12.2 (29,999,923–30,090,863) × 0.855072
chr22:41489001	LOSS	CNV	*EP300*	22q13.2 (41,489,001–41,574,996) × 0.971014

CNV: Copy Number Variation; LOH: Loss of Heterozygosity. * The locus is annotated using GRCh38/hg38 (UCSC Genome Browser, http://genome.ucsc.edu (accessed on 10 March 2025)). ** The standard copy number is 2.

## Data Availability

Data is contained within the article.
